# Characterization of Thermoresponsive Methylcellulose-Based Injectable Hydrogels Incorporating 58S Bioactive Glass for Non-Load-Bearing Bone Regeneration

**DOI:** 10.1021/acsomega.6c00586

**Published:** 2026-05-14

**Authors:** Marina Bosso, Ângela Maria Moraes

**Affiliations:** Department of Engineering of Materials and of Bioprocesses/School of Chemical Engineering (Faculdade de Engenharia Química), University of Campinas (Universidade de Campinas - UNICAMP), Av. Albert Einstein, 13083-852 Campinas, SP, Brazil

## Abstract

Bone fractures represent a significant global public health challenge, particularly in aging populations. Despite extensive research, injectable systems that balance bioactivity with precise handling properties remain scarce. In this study, a novel triple-polymeric blend of methylcellulose, xanthan gum, and pullulan was developed using design of experiments (DoE) to create a multifunctional bioactive carrier for 58S Bioglass. This synergistic matrix was designed for minimally invasive application in non-load-bearing bone regeneration. A comprehensive physicochemical and rheological characterization was performed, as well as preliminary biological screening. The formulations were evaluated for rheological behavior, injectability, mechanical properties, swelling, degradation, thermal stability, morphology, and cytotoxicity. The innovative blend achieved precise gelation near physiological temperatures and high postinjection stability, with viscosity recovery exceeding 80% and pseudoplastic behavior tailored for injectable delivery. Low compressive moduli (∼0.01 kPa) confirmed an ultrasoft nature, prioritizing its role as a localized signaling vehicle over structural support. Degradation studies showed that increasing bioactive glass content modulates mass loss through ionic interactions within the polymeric network. Preliminary biological assays demonstrated no cytotoxicity against L929 mouse fibroblasts and human dental pulp mesenchymal stem cells (DPSCs). Morphological analysis revealed porous, interconnected structures influenced by the bioglass content. Overall, this study establishes the feasibility of a novel hydrogel designed as a versatile and smart platform for minimally invasive bioactive glass delivery in non-load-bearing bone tissue repair.

## Graphical abstract

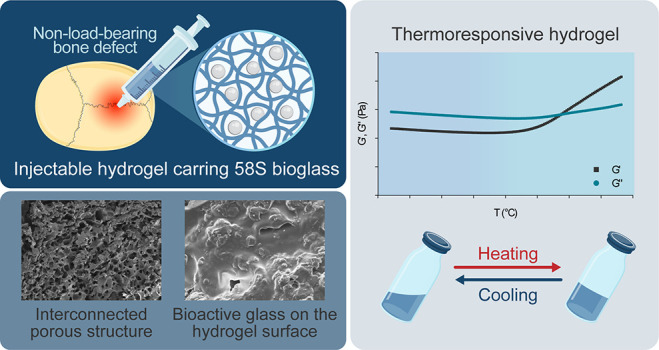

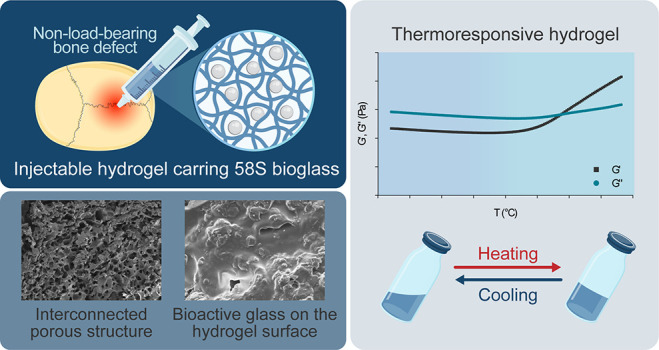

## 1 Introduction

Bone fractures represent a major global public health concern, with substantial clinical and economic impacts, particularly among elderly populations affected by osteoporosis. In 2019, approximately 178 million new fracture cases were reported worldwide, reflecting a 33.4% increase since 1990, with older adults, especially women over 64 years old, being significantly more affected.^1^ In addition to the clinical burden, fracture-related treatments impose substantial economic costs; for example, the average hospital cost of a hip fracture may exceed US$43,000 within one year when comprehensive care is considered.^2^

Large bone fractures and critical-sized defects, especially those associated with trauma or chronic diseases, often require therapeutic strategies capable of actively supporting bone regeneration. Conventional approaches, such as autologous and allogeneic bone grafts, remain the clinical gold standard but are limited by donor-site morbidity, risk of immune rejection, prolonged recovery time, and high costs. These limitations have intensified the search for alternative biomaterial-based strategies, positioning tissue engineering as a promising approach for bone repair and regeneration.^3^

Among the biomaterials explored for bone regeneration, bioactive glasses are distinguished by their capacity to bond with host tissue through the *in situ* formation of a hydroxyapatite-like layer. Since Larry Hench’s seminal development of the quaternary 45S5 composition (45% SiO_2_, 24.5% CaO, 24.5% Na_2_O, and 6% P_2_O_5_ mol) in 1969, alternative formulations incorporating MgO, K_2_O, or B_2_O_3_ have been designed to improve dissolution rates and angiogenic potential.^4,5^ This angiogenic stimulus is particularly critical for larger tissue-engineered constructs (>1 mm), which often face limited nutrient diffusion and waste transport due to the lack of an established vascular network.^6^ The pro-angiogenic properties of bioglass are attributed to the controlled release of silicate, phosphate, and calcium ions, which promote endothelial cell growth, migration, and neovascularization.^6^ While recent strategies have explored doping these structures with metallic ions such as copper or cobalt to further activate pro-angiogenic growth factors, the intrinsic ion release from the bioglass matrix remains a fundamental driver of neovascularization.^6,7^ In this context, the 58S bioglass (BG) stands out as a superior candidate for integration into composite systems. By featuring higher proportions of CaO (36%) and SiO_2_ (58%) relative to the 45S5 benchmark, this formulation promotes faster calcium ion release and accelerated mineralization kinetics, simultaneously supporting the biological signaling required for both osteogenesis and vascular integration.^8^ Furthermore, its enhanced processability ensures a homogeneous dispersion within polymeric phases, providing the essential bioactive cues to the biological environment. However, the effective clinical application of these inorganic particles depends on a specialized vehicle capable of ensuring precise delivery and structural integrity.

Hydrogels fulfill this role as three-dimensional networks of hydrophilic polymers capable of retaining large amounts of water or biological fluids, enabling efficient mass transport and controlled release of bioactive agents. Injectable hydrogels offer additional advantages, including minimally invasive administration and the ability to conform to irregular defect geometries.^9^ In this regard, the combination of thermoresponsive hydrogels with bioactive phases, including hydroxyapatite and bioactive glass, has proven successful in providing both structural support, angiogenesis, and osteoconductive cues.^10–13^

Natural polymers are particularly attractive for these systems due to their biocompatibility and favorable rheological behavior.^9^ In this context, cellulose derivatives, xanthan gum, and pullulan have emerged as promising components for injectable hydrogel formulations.

Methylcellulose (MC) is a thermoresponsive cellulose ether whose water solubility requires a degree of substitution (DS) between 1.3 and 2.5.^14^ Beyond solubility, molar weight (MW) dictates thermal behavior, which high-molar-weight chains undergo gelation well above physiological limits, and low-molar-weight grades (14 kDa version) allow for higher polymer concentrations (up to 16% w/v) and a transition temperature near 31 °C.^15^ This specific combination of structural parameters ensures that the hydrogel maintains a processing window suitable for injection at room temperature while undergoing physically cross-linked gelation *in situ* upon reaching body temperature.

Xanthan gum, a highly water-soluble polyelectrolyte, is characterized by high viscosity at low concentrations and pronounced pseudoplastic behavior, contributing to improved injectability and structural stability in hydrogel systems.^16,17^ Pullulan, a microbial exopolysaccharide, complements these properties by offering high hydration capacity, biocompatibility, and favorable rheological behavior, making it suitable for use in tissue engineering matrices.^18,19^ Although these polymers individually and synergistically exhibit properties desirable for injectable hydrogels, their integration into bioactive systems suitable for bone regeneration remains insufficiently explored.

A recent targeted bibliometric survey (March 3, 2026) across the Scopus, Web of Science, and PubMed databases showed that, by utilizing the keywords “non-load-bearing” and “hydrogel” for the ten-year period from 2016 to 2025, a total of 29 studies were identified after the removal of duplicates. From this selection, ten articles were prioritized for their focus on the development of specialized scaffolds, including conventional hydrogels, cryogels, and 3D-printed structures. These studies revealed a strong trend toward bioactive integration, with nine out of ten formulations incorporating osteoconductive or pro-angiogenic agents to drive tissue repair. Regarding fabrication, three studies focused on 3D-printed or structured scaffolds^20–22^ designed for precise anatomical fitting. Notably, only one of these^20^ relied solely on structural support without the addition of bioactive agents, while the others incorporated hydroxyapatite^22^ or calcium phosphates.^21^ In contrast, only two works explored injectable formulations, targeting minimally invasive procedures by delivering bioactive glass microspheres^23^ or silica nanoparticles.^24^ The remaining five studies described conventional hydrogels or cryogels, which, although bioactive, are limited to preformed or *in situ* cross-linked applications without advanced delivery mechanisms.^25–29^

A critical analysis of this landscape reveals that while bioactivity is well-established, there is a clear technological stagnation regarding smart delivery. The scarcity of injectable and thermoresponsive options among the surveyed materials highlights a significant gap. Most current research of non-load-bearing hydrogels focuses either on complex 3D architecture or bioactivity, leaving a gap for biomaterials that can combine injectability, thermoresponsivity *in situ* at body temperature, and targeted signaling in a single cohesive system.

Therefore, the present study aimed to develop and comprehensively characterize a novel injectable thermoresponsive hydrogel based on a synergistic blend of methylcellulose, xanthan gum, and pullulan enriched with 58S bioglass. This investigation focused on the physicochemical, rheological, and mechanical performance of this composite hydrogel platform, coupled with a preliminary cytocompatibility screening to evaluate its suitability as a smart carrier system for non-load-bearing bone repair. The novelty of this work lies in the strategic integration of these three natural polysaccharides to overcome the limitations of rigid scaffolds, resulting in a dual-action system in which the matrix provides thermoresponsive delivery while the 58S bioglass acts as both a structural modulator and a bioactive agent. By establishing this multifunctional platform, this work proposes a biomaterial capable of providing minimally invasive delivery and possible localized bioactivity, addressing key technological limitations in the development of injectable constructs for bone tissue engineering.

## 2 Materials and Methods

### 2.1 Materials

Methylcellulose (MC; viscosity 15 cP, average molar weight around 14 kDa, with degree of substitution between 1.5 and 1.9; batch no. 318699), xanthan gum (XG; viscosity in the range 800 to 1200 cP; batch no. 368682), and 58S bioglass (BG, batch nos. MKCW0372 and MKCW3697) were purchased from Sigma-Aldrich (USA). Pullulan (PUL; batch no. WIT2025032809) was obtained from Pratic (Brazil). Glycerol (GL, ACS grade; batch no. 202209953-1L) was purchased by ACS Científica (Brazil). Phosphate-buffered saline (PBS, pH 7.4; batch no. 78125205) was from Invitrogen (USA). All other reagents were of at least analytical grade.

### 2.2 Methods

#### 2.2.1 Preparation of Hydrogels

The hydrogels were prepared following a previously reported procedure,^30^ as schematically shown in Figure 1. Briefly, stock solutions of MC (15% w/v), XG (10% w/v), and PUL (10% w/v) were prepared in PBS, at pH 7.4 under mechanical stirring at 600 rpm and room temperature. For the xanthan gum solution, glycerol (5% w/v) was incorporated as a wetting agent to ensure proper polysaccharide hydration and homogeneous dispersion, preventing the formation of insoluble aggregates.^31^ The selection of this concentration took into account the established toxicity limit of glycerol at 20% (v/v).^32^ From a structural perspective, the three hydroxyl groups of glycerol establish extensive intermolecular hydrogen bonding with the hydrophilic carboxyl and hydroxyl groups of the xanthan chains, as well as with surrounding water molecules. By intercalating between XG chains, glycerol modulates the hydration shell and competes with direct polymer–polymer self-association by hydrogen bonding. This mechanism effectively reduces the formation of aggregates during initial dissolution, while also slightly influencing weak polymer hydrophobic interactions by altering the solvent’s dielectric microenvironment,^33^ ultimately leading to a more uniform and stable polymeric solution.

**Figure 1:**
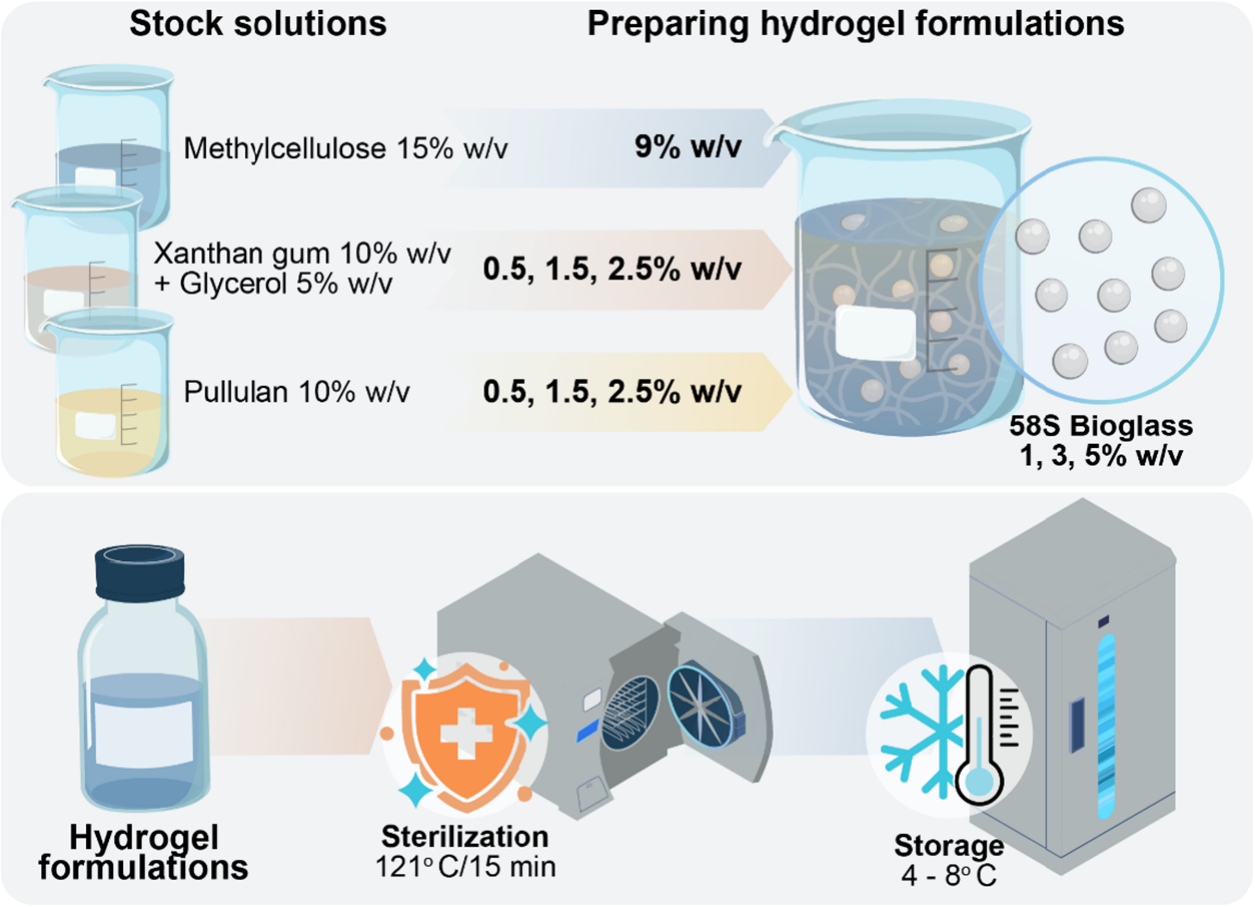
Schematic diagram showing the production stages of the hydrogel formulations (figure designed by Linear Scientific Visual Communication company under the author’s direction).

To avoid processing limitations due to the high viscosity of methylcellulose stock solution in PBS, the polymer was solubilized at low temperatures (4–8 °C) to prevent premature thermal gelation and ensure complete dissolution. The resulting solution was stored at 4 °C for 24 h to stabilize.

Subsequently, the polymer stock solutions were diluted with PBS to reach the target concentrations defined in the experimental design and homogenized at 600 rpm. 58S bioglass was then incorporated into the polymeric mixtures under continuous stirring, based on the parameters established by Zeimaran and colleagues.^9^ Finally, the resulting hydrogels were sterilized in an autoclave at 1 atm and 121 °C for 15 min and stored at 4 °C for 24 h before characterization.

To systematically evaluate the influence of the formulation components on the performance of the hydrogel, a 2^3^ full factorial experimental design was employed, including a central point, and all conditions were analyzed in triplicate. This statistical approach allowed for the simultaneous investigation of the effects of three independent variables (xanthan gum, pullulan, and 58S bioglass) at two distinct levels. These levels are represented in a coded matrix as −1 (lower level) and +1 (upper level), a normalization procedure that enables the unbiased comparison of factors with different physical magnitudes and the identification of synergistic or antagonistic interactions (Table 1).

**Table 1:** Full Factorial Experimental Design (2^3^) with Central Points, with Coded and Uncoded Formulations, Describing Varying and Non-Varying Components

	**coaded design matrix**				**actual uncoaded concentration values**				
	**levels of concentration of varying components** ^a^				**concentration of fixed components** ^b^		**concentration of varying components** ^c^		
**experiment**	**XG**	**PUL**	**BG**	**formulation description**	**MC (% m/v)**	**GL (% m/v)**	**XG (% w/v)**	**PUL (% w/v)**	**BG (% w/v)**
1	–1	–1	–1	MC9XG0.5PUL0.5BG1-GL5	9	5	0.5	0.5	1
2	+1	–1	–1	MC9XG2.5PUL0.5BG1-GL5			2.5	0.5	1
3	–1	+1	–1	MC9XG0.5PUL2.5BG1-GL5			0.5	2.5	1
4	+1	+1	–1	MC9XG2.5PUL2.5BG1-GL5			2.5	2.5	1
5	–1	–1	+1	MC9XG0.5PUL0.5BG5-GL5			0.5	0.5	5
6	+1	–1	+1	MC9XG2.5PUL0.5BG5-GL5			2.5	0.5	5
7	–1	+1	+1	MC9XG0.5PUL2.5BG5-GL5			0.5	2.5	5
8	+1	+1	+1	MC9XG2.5PUL2.5BG5-GL5			2.5	2.5	5
9	0	0	0	MC9XG1.5PUL1.5BG3-GL5			1.5	1.5	3

a –1: lower level; +1: upper level; 0: central point condition.

b MC: methylcellulose; GL: glycerol.

c XG: xanthan gum; PUL: pullulan; BG: 58S bioglass.

The uncoded matrix, which provides the actual concentrations in mass to volume percentages corresponding to the lower and upper levels, is detailed alongside the coded values and the identification of the formulations in Table 1. The inclusion of a central point (level 0) is critical to assess experimental reproducibility and to detect potential nonlinear behaviors (curvature) within the explored experimental domain. While the concentrations of XG, PUL and BG were varied, the concentrations of MC (9%) and GL (5%) were maintained constant, based on previous optimization by Westin and colleagues,^30^ which identified these specific concentrations as ideal for ensuring thermoresponsive behavior and stable XG dispersion without compromising the structural integrity of the hydrogels.

#### 2.2.2 Qualitative and Quantitative Temperature Gelation Analysis

Qualitative analysis of thermoresponsive gelation and physical cross-linking of the hydrogels was assessed using the tube inversion method. Formulations (3 mL) were sequentially incubated at 22, 37, and 4 °C; following each stage, the tubes were inverted 180° for 5 min to evaluate flow behavior. Photographic records were used to verify the expected transition from a fluid state at 22 °C (simulating the clinical injection process) and 4 °C (simulating storage temperature), to a nonflowing gel state at 37 °C (mimicking physiological conditions).^34^

Quantitative determination of the gelation temperature was performed using oscillatory rheological analysis in an Anton Paar MCR 102 rheometer equipped with a cone–plate geometry. Temperature sweep tests were conducted from 4 to 40 °C at a heating rate of 1 °C·min^–1^, under constant stress (5 Pa) and angular frequency (10 rad·s^–1^). The gelation temperature was defined as the crossover point at which the storage modulus (*G*′) exceeded the loss modulus (*G*″), indicating the transition from viscous to gel-like behavior.^30^

#### 2.2.3 Injectability Test

Injectability of the hydrogels was evaluated using a texture analyzer (TA.XT Plus, Stable Micro Systems) equipped with a 500 N load cell and a syringe extrusion holder. The assays were performed using 10 mL disposable syringes coupled or not to sterile, lubricated 18G needles (internal diameter 0.84 mm, length 40 mm), to simulate injection in small or large lesions. Each syringe was loaded with 5 mL of hydrogel and tested at 22 °C, which represented the upper limit of the temperature range for surgical centers (18–22 °C) established by the Brazilian National Health Surveillance Agency (ANVISA),^35^ while also falling within the 20–24 °C range recommended by the FDA and EMA for operating rooms.^36^ A preload of 0.15 N was applied before extrusion, after which the syringe plunger was compressed at a constant speed of 4.0 mm·s^–1^ up to a maximum displacement of 10 mm.

#### 2.2.4 Indirect Cytotoxicity of the Hydrogel Formulations

Indirect cytotoxicity was evaluated according to ISO 10993-5 ^37^ using L929 mouse fibroblasts and human dental pulp stem cells (hDPSCs, from Lonza), both kindly provided by Prof. Dr. Ibsen Bellini Coimbra (School of Medical Sciences/University of Campinas). Cells were seeded in 96-well plates (10^4^ cells/well) and cultured in DMEM medium (from Gibco) supplemented with 10% (v/v) fetal bovine serum, 100 U/mL penicillin, and 100 μg/mL streptomycin at 37 °C and 5% CO_2_ for 24 h. Hydrogel extracts were prepared by incubating samples of the sterilized formulations in culture medium (0.1 g/mL) for 24 h at 37 °C. Subsequently, cells were exposed to these extracts for 24 h, and metabolic activity was assessed via the MTT assay, with absorbance measured at 570 nm (Varioskan LUX, Thermo Fisher Scientific). Triton X-100 (1%) and supplemented medium were used as positive and negative controls, respectively. Cell viability was calculated according to eq 1:

1 $$\begin{array}{l} \mathrm{cell}\;\mathrm{viability}\;\%=\frac{100\times {\mathrm{OD}}_{570e}}{{\mathrm{OD}}_{570b}} \end{array}$$

where OD_570e_ is the average absorbance value of the samples incubated with the extracts and OD_570b_ is the average absorbance value of the samples from the negative control assays. In accordance with ISO 10993-5, formulations resulting in viability above 70% are not considered potentially cytotoxic.

#### 2.2.5 Surface Morphology

Microstructural and morphological analyses of the hydrogels were performed using scanning electron microscopy (SEM) at an accelerating voltage of 15 kV. To evaluate the impact of thermal transition on the matrix architecture, samples were prepared under two distinct conditions: (i) non-cross-linked (maintained at 22 °C) and (ii) thermally cross-linked (incubated at 37 °C for 30 min). All specimens were frozen in an ultrafreezer at −70 °C for 24 h, lyophilized for 72 h under a vacuum of 720 mmHg at a temperature of −41 °C, and stored in a desiccator before analysis. In accordance with ASTM F2450,^38^ and to overcome the inherently low electron density of polymeric scaffolds, samples intended for cross-sectional imaging were subjected to conductive coating. This was achieved by applying a graphite suspension dispersed in isopropanol. After complete drying, the samples were mounted on metallic stubs, and the morphology was examined at a representative magnification.

#### 2.2.6 Rheology Analysis

Rheological properties of the hydrogel formulations were evaluated using a modular rheometer (Anton Paar MCR 102) equipped with a cone–plate geometry. Measurements were conducted at 20 °C to simulate the average room temperature of surgical environments, thus representing the behavior of the biomaterial during clinical handling.^35^ Flow behavior was assessed by measuring viscosity as a function of shear rate (γ̇), ranging from 0.1 to 1000 s^–1^, to determine whether the hydrogels exhibited pseudoplastic, dilatant, or viscoplastic behavior. Amplitude sweep tests were conducted over a strain range of 0.01 to 10,000% at a constant angular frequency of 10 rad·s^–1^ to determine the viscoelastic behavior. Frequency sweep measurements were performed within the LVE (γ = 1%), over an angular frequency range from 100 to 0.1 rad·s^–1^.

Thixotropic behavior and viscosity recovery were evaluated using a three-interval thixotropy test (3ITT), consisting of: (i) low shear rate (γ̇ = 1 s^–1^) for 120 s to establish initial viscosity; (ii) high shear rate (γ̇ = 100 s^–1^) for 60 s to simulate injection; and (iii) recovery phase at low shear rate (γ̇ = 1 s^–1^) for 60 s. The viscosity recovery percentage (VR) was calculated according to eq 2:

2 $$\begin{array}{l} VR\;\left(\%\right)=\left(\frac{\eta_{t\left(3\right)}}{\eta_{t\left(1\right)}}\right)\times 100 \end{array}$$

in which VR represents the viscosity recovery percentage, and η_t(3)_ and η_t(1)_ are the viscosities in steps (3) and (1), respectively.

#### 2.2.7 Compressive Test

The mechanical properties of the hydrogels were evaluated by unconfined compression tests using a texture analyzer (TA.XT Plus, Stable Micro Systems) equipped with a 50 N load cell. Hydrogel samples were prepared in 24-well plates by dispensing 1 mL of each formulation per well, followed by gelation at 37 °C for 30 min. Cylindrical specimens (13–20 mm diameter) were subjected to compression tests at room temperature (22 °C), using a constant crosshead speed of 0.1 mm·s^–1^ up to a maximum strain of 70%.^39^ Stress–strain curves were recorded, and the Young’s modulus was determined from the slope of the linear elastic region of the curve.^39^

#### 2.2.8 Swelling and Degradation

The swelling behavior of the hydrogels was evaluated by incubating samples (0.2 g) in PBS (pH 7.2) at 37 °C. Each sample was placed in a 1.5 mL microtube containing 0.8 mL of PBS and incubated for 24 h. After incubation, excess surface liquid was gently removed, and the swollen hydrogels were weighed. The swelling degree (%) was calculated according to eq 3:

3 $$\begin{array}{l} \mathrm{swelling}\;\mathrm{degree}\;\left(\%\right)=\frac{\left(W_{s}-W_{i}\right)}{W_{i}}\times 100 \end{array}$$

where *W_s_* is the mass of the swollen hydrogel, and *W*_*i*_ refers to its initial mass.

*In vitro* degradation was assessed by incubating hydrogel samples (0.2 g) in 0.8 mL of PBS at 37 °C for up to 30 days. The PBS medium was periodically refreshed every 48–72 h. At predetermined time points (5, 10, 15, and 30 days), samples were removed, frozen at – 70 °C, and lyophilized. The mass loss (%) was calculated as

4 $$\begin{array}{l} \mathrm{degradation}\;\left(\%\right)=\frac{\left(W_{d}\left(0\right)-W_{d}\left(t\right)\right)}{W_{d}\left(0\right)}\times 100 \end{array}$$

where *W*_*d*_(0) is the initial mass of the dry material and *W*_*d*_(*t*) is the mass of the dried hydrogel at time *t*.

#### 2.2.9 Infrared Spectroscopy and Thermogravimetric Analyses

Physicochemical characterization of the formulations was performed by using Fourier Transform Infrared (FTIR) spectroscopy and thermogravimetric analysis (TGA).

FTIR was carried out (Agilent Cary 660) to assess the chemical interactions between the polymeric matrix and the bioactive glass. Lyophilized samples were prepared by freezing at −70 °C for 24 h, followed by lyophilization for 72 h before analysis. Detailed results and spectral analysis are provided as Supporting Information.

TGA was performed as standard thermal characterization. Lyophilized hydrogel samples were analyzed (TGA/DSC1 analyzer, Mettler Toledo, Switzerland) under a nitrogen atmosphere. The measurements were conducted over a range of 25 to 500 °C, at a heating rate of 20 °C·min^–1^. Detailed results and thermogravimetric curves are provided as Supporting Information.

#### 2.2.10 Statistical Analysis

Numerical data were expressed as mean ± standard deviation. For the formulation screening study, main effects and interaction effects of the formulation variables were evaluated at 95% confidence level (*p* ≤ 0.05) using the Statistica software (version 7) to determine their statistical significance and influence on the measured responses. Data normality was assessed using the Shapiro–Wilk test for each quantitative variable within the experimental groups. When normality assumptions were met, differences among group means were evaluated by one-way analysis of variance (ANOVA), followed by Tukey’s post hoc test, with a significance level set at 5% (*p* ≤ 0.05). These statistical analyses were performed using the OriginPro software (version 8.5).

## 3 Results and Discussion

### 3.1 Selection of Adequate Formulations Regarding Gelation Temperature and Injectability

Through direct visual analysis of the formulations exposed subsequently to 22, 37, and 4 °C, it was possible to observe that all hydrogels remained homogeneous throughout the thermal cycles (Figure S1). The qualitative tube inversion assays showed that at 37 °C, all formulations were in the nonflowing gel state. At 23 °C, formulations with higher xanthan gum content (1.5–2.5 wt %) already showed signs of initial gelation, whereas cooling to 4 °C restored their flowability.

Temperature-sweep gelation and extrusion force assays were employed as preliminary screening tools to select hydrogel formulations for further characterization. Rheological temperature-sweep results demonstrated that formulations containing higher xanthan gum concentrations (1.5–2.5 wt %) exhibited gelation temperatures below or close to 23 °C (Figure 2).

**Figure 2:**
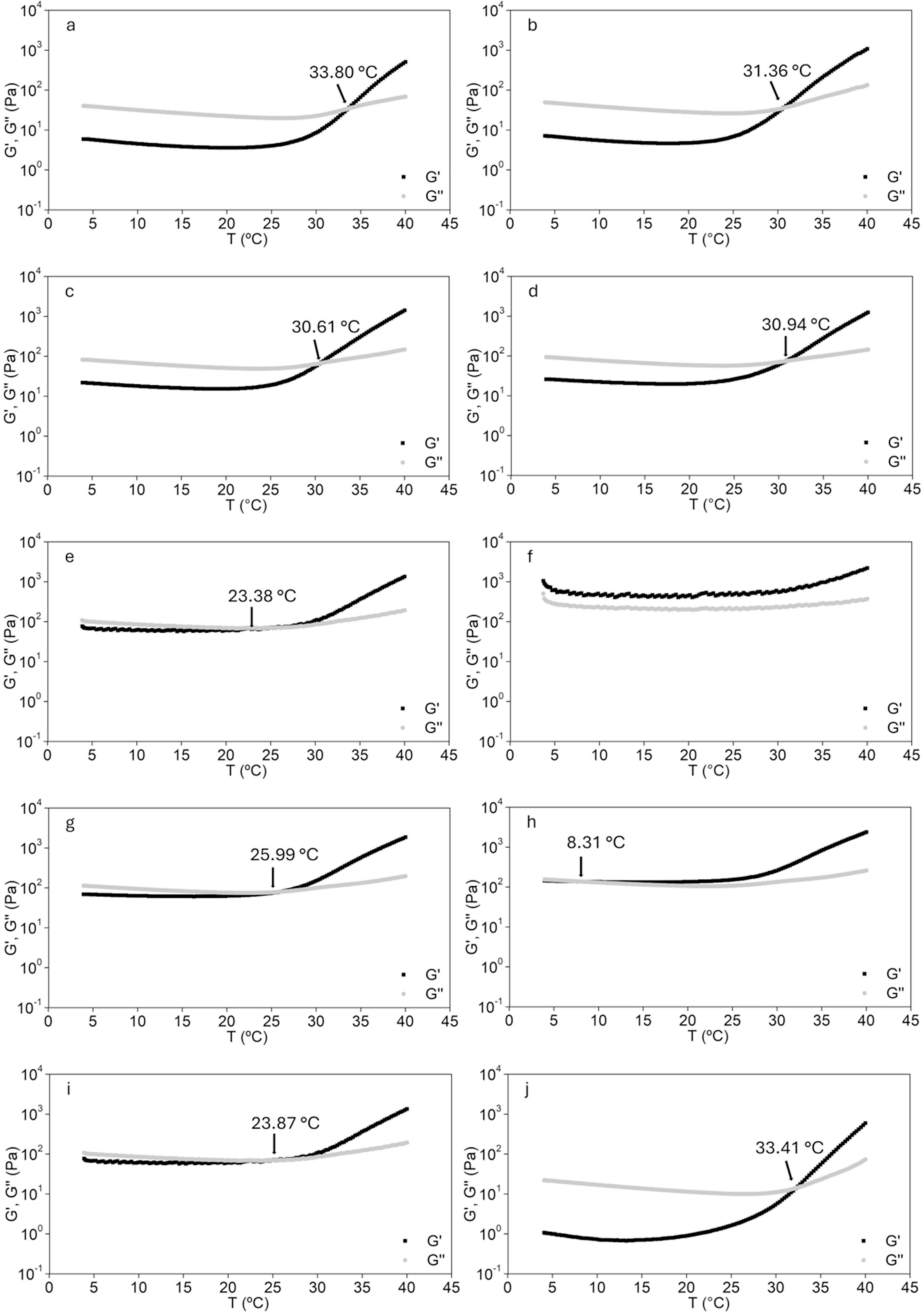
Storage (*G*′) and loss (*G*″) moduli as a function of temperature for the different hydrogel formulations: (a) MC9XG0.5PUL0.5BG1-GL5; (b) MC9XG0.5PUL0.5BG5-GL5; (c) MC9XG0.5PUL2.5BG1-GL5; (d) MC9XG0.5PUL2.5BG5-GL5; (e) MC9XG2.5PUL0.5BG1-GL5; (f) MC9XG2.5PUL0.5BG5-GL5; (g) MC9XG2.5PUL2.5BG1-GL5; (h) MC9XG2.5PUL2.5BG5-GL5; (i) MC9XG1.5PUL1.5BG3-GL5; (j) methylcellulose 9% (w/v).

According to Brazilian, FDA, and EMA regulatory guidelines, operating room temperatures are typically maintained between 18 and 24 °C.^35,36^ Therefore, formulations that gelled within or below this range were considered unsuitable for injectable handling and were excluded from subsequent analyses.

Statistical evaluation of gelation temperature data by ANOVA followed by Tukey’s test revealed significant differences primarily associated with xanthan gum concentration, regardless of the levels of pullulan or bioactive glass. Additionally, formulations containing simultaneously high xanthan gum (2.5 wt %) and high bioglass content (5 wt %) showed significantly lower gelation temperatures compared to those with equivalent xanthan gum content and lower glass loading (1 wt %), indicating a relevant interaction effect between xanthan gum and bioglass at higher concentrations. Effect analysis at a 95% confidence level confirmed xanthan gum and bioglass concentrations as significant main factors, as well as their interaction and the interaction between xanthan gum and pullulan.

The reduction in gelation temperature observed at higher xanthan gum contents can be attributed to intermolecular dehydration interactions between the carboxyl groups of xanthan gum and the methyl groups of methylcellulose, which promote earlier network formation.^40^ Furthermore, the presence of divalent cations released from the bioglass and from PBS partially shields the anionic charges of xanthan gum, reducing electrostatic repulsion and facilitating closer polymer chain packing. This structural rearrangement favors gel network formation at lower temperatures, explaining the synergistic effect observed for formulations with high xanthan gum and bioactive glass contents.^17,18^

Extrusion force measurements demonstrated that in both conditions, with and without an 18G needle, increasing xanthan gum and bioactive glass concentrations led to a significant rise in extrusion force (Table 2). ANOVA results confirmed statistically significant differences among formulations, with xanthan gum emerging as the dominant factor influencing extrusion resistance.

**Table 2:** Mean Values of the Maximum Force Required to Extrude Each Hydrogel Formulation, with and without an 18G Needle^a^

**hydrogel**	**mean maximum extrusion force (N) without needle**	**mean maximum extrusion force (N) (18G)**
MC9XG0.5PUL0.5BG1-GL5	4.955 ± 0.301^a^	69.284 ± 3.762^a^
MC9XG0.5PUL0.5BG5-GL5	5.673 ± 0.220^a^	82.621 ± 4.645^a,b^
MC9XG0.5PUL2.5BG1-GL5	5.903 ± 0.220^a^	90.462 ± 6.542^b,c,d^
MC9XG0.5PUL2.5BG5-GL5	6.239 ± 0.632^a^	95.448 ± 3.664^b,c,d^
MC9XG2.5PUL0.5BG1-GL5	13.240 ± 0.201^c^	87.172 ± 5.369^a,b^
MC9XG2.5PUL0.5BG5-GL5	20.943 ± 0.549^b^	107.107 ± 11.371^d^
MC9XG2.5PUL2.5BG1-GL5	16.585 ± 1.348^d^	127.695 ± 10.460^e^
MC9XG2.5PUL2.5BG5-GL5	19.050 ± 1.082^b^	147.092 ± 0.899^f^
MC9XG1.5PUL1.5BG3-GL5	11.258 ± 1.564^c^	82.655 ± 0.315^a,b^

a The same letter in the same column indicates that there is no significant difference between the mean values (Tukey’s test, *p* < 0.05).

Xanthan gum had the most substantial influence on extrusion force across all tested conditions. In the absence of a needle, formulations with 0.5 wt % xanthan gum required lower forces regardless of other components, although bioactive glass concentration emerged as a secondary modulator at higher xanthan levels. While xanthan gum and bioglass were the predominant factors in needle-free tests, the introduction of the 18G needle shifted this dynamic by significantly increasing the relevance of pullulan. Under these confined shear conditions, the contribution of pullulan surpassed that of bioglass, which is likely due to the specific rheological resistance this polymer offers within the narrow geometry of the needle.

Statistical analysis further revealed that xanthan gum interacted significantly with bioglass in the open syringe system, whereas its interaction with pullulan was significant only during needle-assisted extrusion. In contrast, no significant interaction between bioglass and pullulan was detected under any condition. Collectively, the increased resistance observed at higher xanthan gum and bioglass concentrations is consistent with the formation of a dense polymeric network stabilized by ionic cross-linking between xanthan gum carboxylate groups and divalent cations (Ca^2+^).^41,42^ These results underscore that injectability must be evaluated under application-specific geometries because needle-induced confinement fundamentally alters the mechanical contribution of each polymeric component.

Hydrogels extruded without the 18G needle required forces below 30 N, indicating excellent manual injectability, according to thresholds for comfortable manual administration reported in the literature.^43^ In contrast, extrusion forces using 18G needles ranged from approximately 70 to 150 N, suggesting that practical application would require either temperature control before injection or the use of specialized syringes commonly employed in orthopedic procedures. Such systems, including screw-driven syringes or high-pressure applicator guns, are designed to provide the mechanical advantage required to deliver high-viscosity biomaterials steadily and precisely into bone defects, ensuring safety and ease of handling during surgical procedures.

Based on the combined analysis of gelation temperature and extrusion force, two formulations were selected for further analysis, as the most promising: MC9XG0.5PUL2.5BG1-GL5 and MC9XG0.5PUL2.5BG5-GL5. These formulations present reduced xanthan gum content (identified as the most influential factor on both gelation temperature and injectability), higher pullulan content, and varying bioactive glass concentrations, allowing assessment of the specific contribution of the bioactive phase while maintaining suitable handling properties. For comparison purposes, formulations without bioactive glass were also analyzed.

### 3.2 Characterization of the Most Promising Candidate Formulations

#### 3.2.1 Indirect Cytotoxicity

The cell viability of L929 mouse fibroblasts and human dental pulp stem cells (hDPSCs) was evaluated after 24 h of exposure to hydrogel extracts using the MTT assay. According to ISO 10993-5 ^37^ standards, biomaterials are considered noncytotoxic if cell viability remains above 70% relative to the negative control. As shown in Figure 3, all formulations, regardless of the bioactive glass content, supported metabolic activities well above this threshold, confirming the noncytotoxicity of the developed injectable systems.

**Figure 3:**
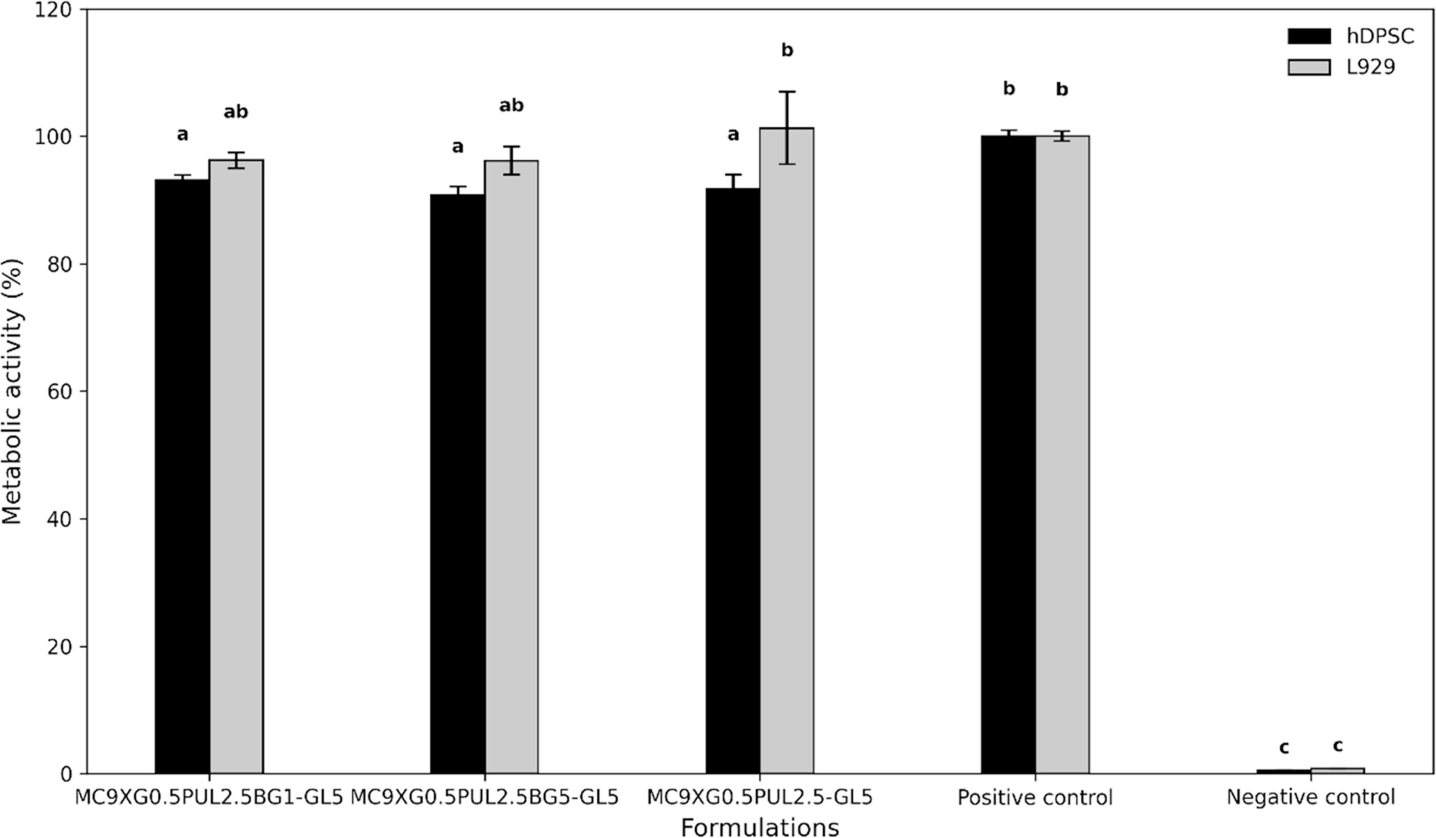
Metabolic activity (MTT assay) of L929 fibroblasts and hDPSCs cultured for 24 h with extracts from hydrogel formulations with and without bioactive glass. Identical letters indicate no significant difference between mean values (Tukey’s test, *p* < 0.05).

Statistical analysis (ANOVA) revealed that viability levels were generally consistent across both cell lines, with no significant differences between L929 and hDPSCs, except for the formulation free of bioglass, which exhibited a distinct response between the two types. Furthermore, while hDPSCs showed statistically significant differences compared to the positive control, L929 cells demonstrated higher resistance, showing no such variation. These findings are consistent with the work of Westin et al.,^30^ who developed noncytotoxic methylcellulose/xanthan gum/carboxymethyl chitosan hydrogels in which metabolic activities exceeding 70% was observed for hDPSCs over 24 and 48 h, confirming the safety of methylcellulose-based matrices.

The incorporation of 58S bioglass did not negatively affect the biological performance of the system. This observation aligns well with the results reported by Malik et al.,^44^ in which injectable carriers based on Pluronic F127 and hydroxypropyl methylcellulose containing bioactive glass maintained MC3T3-E1 preosteoblast viability above 70%, with a gradual increase in proliferation over 7 days.

In summary, the viability percentages obtained here demonstrate that these hydrogel formulations are comparable to state-of-the-art systems in the literature, positioning them as promising noncytotoxic candidates for minimally invasive bone tissue engineering.

#### 3.2.2 Surface Morphology

Morphological analysis was conducted to investigate the superficial and internal microstructure of the hydrogels, comparing cross-linked and non-cross-linked formulations, with and without bioactive glass. Lyophilized samples were used for scanning electron microscopy (SEM) analysis.

Superficial SEM images are presented in Figure 4, and cross-sectional images are presented in Figure 5.

**Figure 4:**
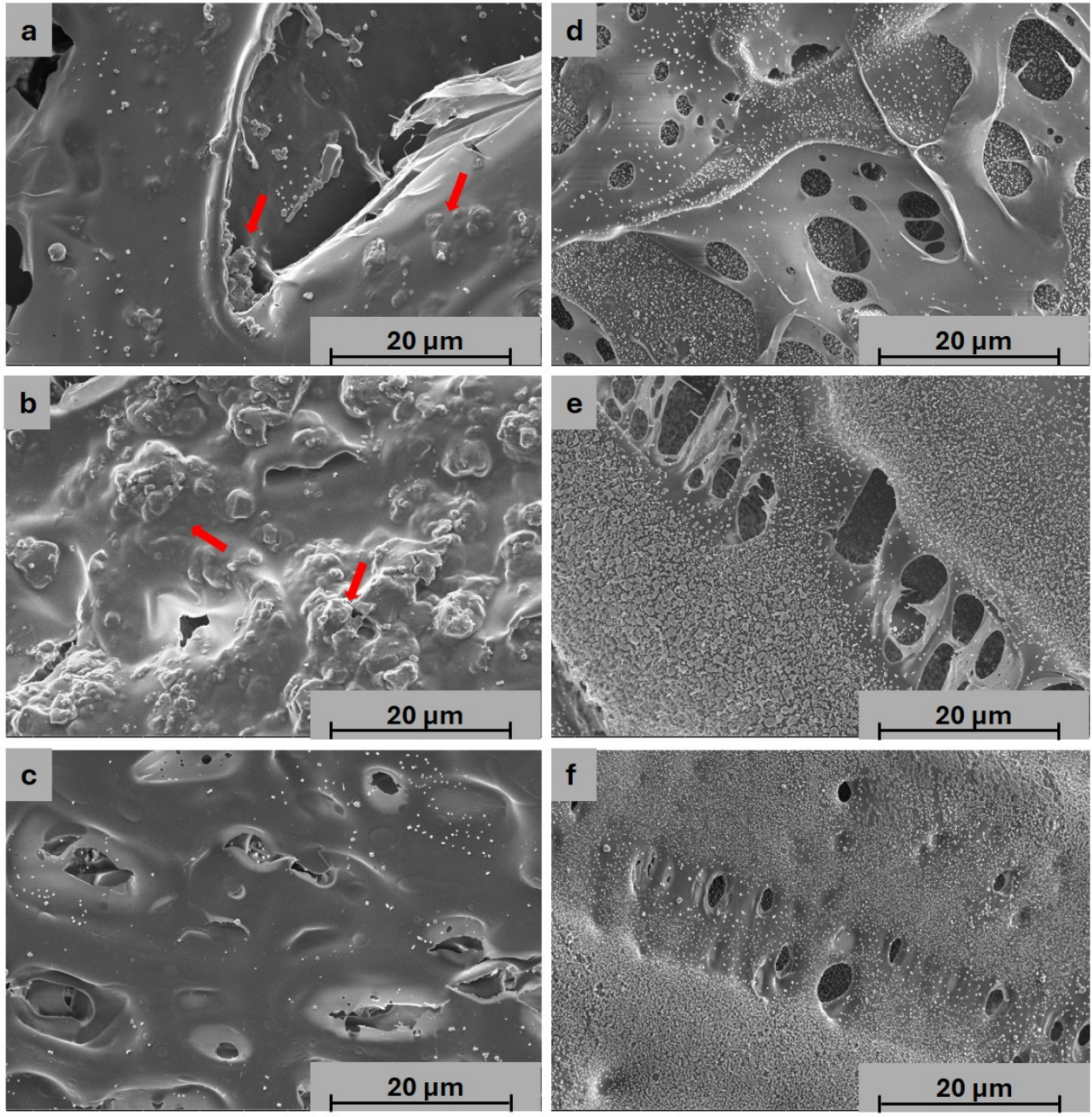
Scanning electron micrographs (SEM) of the surface for cross-linked formulations on the left: (a) MC9XG0.5PUL2.5BG1-GL5, (b) MC9XG0.5PUL2.5BG5-GL5, (c) MC9XG0.5PUL2.5-GL5; and non-cross-linked formulations on the right: (d) MC9XG0.5PUL2.5BG1-GL5, (e) MC9XG0.5PUL2.5BG5-GL5, (f) MC9XG0.5PUL2.5-GL5. Red arrows indicate bioactive glass aggregates in the cross-linked samples.

**Figure 5:**
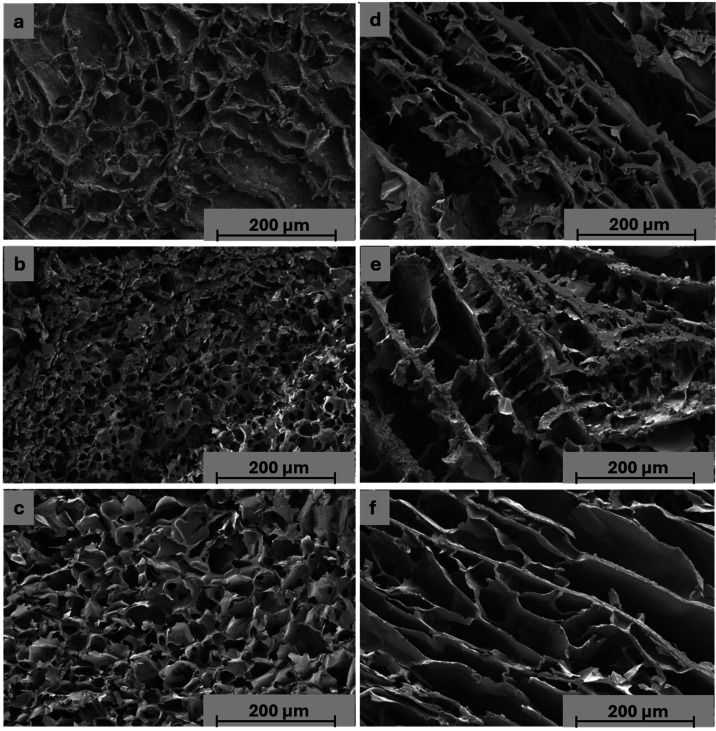
Scanning electron microscopy of the cross-section of the cross-linked formulations on the left (a) MC9XG0.5PUL2.5BG1-GL5; (b) MC9XG0.5PUL2.5BG5-GL5; (c) MC9XG0.5PUL2.5-GL5 and non-cross-linked formulations on the right (d) MC9XG0.5PUL2.5BG1-GL5; (e) MC9XG0.5PUL2.5BG5-GL5; (f) MC9XG0.5PUL2.5-GL5.

The morphological analysis of the superficies identified interconnected pores and, as illustrated in Figure 4, the presence of bioactive glass aggregates (red arrows) in cross-linked formulations, corroborated by EDS detection of Si consistent with the 58S bioglass composition (58% SiO_2_, 36% CaO, 6% P_2_O_5_ in molar fraction). Conversely, 58S bioglass particles were not discernible on non-cross-linked surfaces or in any cross-sectional views. This suggests that non-cross-linked matrices promote an open-mesh structure, which may facilitate the dispersion and embedding of the bioactive phase, hindering its localized identification via SEM/EDS.

SEM micrographs of the cross sections of the hydrogels revealed a heterogeneous and highly interconnected porous morphology, a key feature for ensuring nutrient diffusion and cell infiltration in tissue engineering. The apparent pore sizes ranged from 18 to 30 μm (Table 3). However, these values represent the material in its lyophilized state. As widely reported,^45–47^ freezing and lyophilization processes, followed by high-vacuum analysis such as the SEM technique employed, can induce structural artifacts such as pore wall collapse or expansion due to ice crystal growth. Nevertheless, this classic characterization remains essential for observing structural trends and the successful homogeneous dispersion of particles and fibers within hydrogel polymeric matrices.

**Table 3:** Average Pore Size of Cross-Linked Formulations: MC9XG0.5PUL2.5BG1-GL5; MC9XG0.5PUL2.5BG5-GL5; MC9XG0.5PUL2.5-GL5^a^

**cross-linked formulation**	**average pore size (μm)**
MC9XG0.5PUL2.5BG1-GL5	30.111 ± 7.854^a^
MC9XG0.5PUL2.5BG5-GL5	18.283 ± 4.604^b^
MC9XG0.5PUL2.5-GL5	26.869 ± 9.482^a^

a The same letter in the same column indicates that there is no significant difference between the mean values (Tukey’s test, *p* < 0.05).

Pore size measurements were performed only for the cross-linked formulations. Non-cross-linked samples exhibited an undefined morphology without well-defined pores suitable for quantitative analysis (Table 3), likely due to higher chain mobility and lack of structural constraints in the absence of cross-linking.

Among the cross-linked hydrogels, increasing the bioactive glass content resulted in a more compact microarchitecture and a reduction in average pore size, with the formulation incorporating 5% bioglass presenting the smallest pores. No significant difference in pore size was observed between the formulation without bioglass and that containing 1% bioglass, likely due to the low concentration of the filler relative to the total composition, which may have been insufficient to promote measurable changes in the hydrogel microarchitecture. This dose-dependent trend is consistent with findings by Sarker et al.,^48^ who observed increased structural compactness in bioglass-containing hydrogels. In the present system, this behavior is attributed to ionic interactions between divalent cations (Ca^2+^) released from 58S bioglass and the carboxylate groups of xanthan gum, promoting network contraction and a denser framework.

In this context, even considering that lyophilization and vacuum analysis results may not fully represent absolute pore dimensions, it is reasonable to infer that the developed cross-linked hydrogels possess a highly functional architecture. The constricted and reinforced framework, directly influenced by the ionic cross-linking promoted by the 58S bioglass presence, provides a stable yet dynamic template for cell infiltration and signaling. Ultimately, the synergy between the swelling capacity of the matrix and its degradability ensures that this initial microstructure will evolve physiologically, supporting tissue ingrowth as the polymeric network expands and gradually degrades.

#### 3.2.3 Rheological Behavior

Rheological analyses were performed to evaluate the pseudoplastic, viscoelastic, and thixotropic behavior of the hydrogel formulations at 20 °C. Viscosity measurements as a function of shear rate revealed non-Newtonian behavior for all samples, with a marked decrease in apparent viscosity as the shear rate was increased, characterizing pseudoplastic materials (Figure 6A).^49^ This response is attributed to the progressive disruption of the physically cross-linked polymer network and the release of the entrapped fluid, which is advantageous for injectable systems by facilitating flow during administration and partial structural recovery after shear cessation.^50^

**Figure 6:**
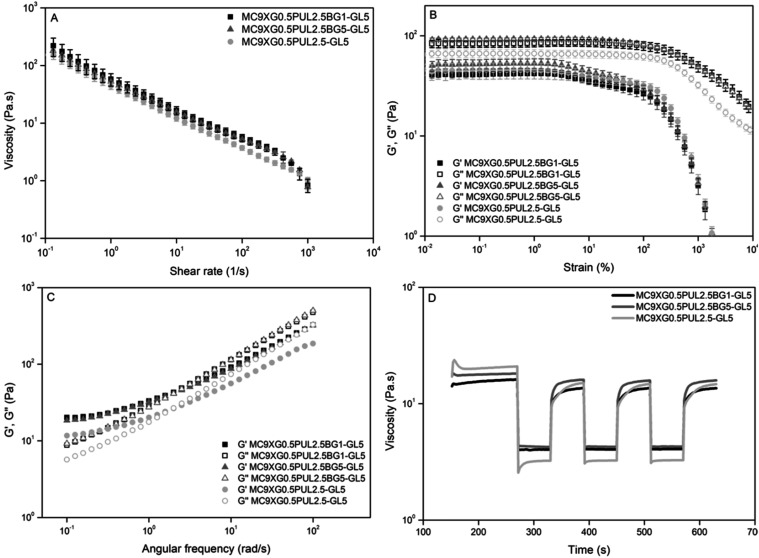
Rheological characterization of the hydrogel formulations MC9XG0.5PUL2.5BG1-GL5, MC9XG0.5PUL2.5BG5-GL5, and MC9XG0.5PUL2.5-GL5: (A) viscosity as a function of shear rate; (B) amplitude sweep; (C) frequency sweep; and (D) three-interval thixotropy test (3ITT).

Amplitude sweep tests were conducted to determine the viscoelastic behavior. In the three formulations, no flow point was identified by the intersection between *G*′ and *G*″, indicating predominantly fluid-like structure and viscoelastic liquid behavior (Figure 6B).^49,50^ Based on the amplitude results, frequency sweep analyses were subsequently performed (Figure 6C). In all formulations, the storage modulus (*G*′) remained higher than the loss modulus (*G*″) at low frequencies, indicating a predominantly elastic behavior and the presence of a structured physical network.^49,51^ A frequency-dependent increase in both moduli was observed, followed by a crossover at higher frequencies. This profile is characteristic of physically cross-linked “weak gels”, in which the stability of the network is maintained by reversible junctions, such as hydrogen bonding and hydrophobic interactions, which exhibit a limited relaxation time scale when subjected to high-frequency oscillations.^52^

Thixotropic recovery was evaluated using a three-interval thixotropy test (3ITT). All formulations exhibited a sharp decrease in viscosity under high shear conditions, followed by partial recovery upon returning to low shear (Figure 6D). Notably, hydrogels containing bioactive glass showed enhanced viscosity recovery, reaching 84.8% for MC9XG0.5PUL2.5BG1-GL5 and 88.8% for MC9XG0.5PUL2.5BG5-GL5, compared to 72.3% for the bioactive-glass-free control (Figure 6D). Recovery values above 80% are considered suitable for injectable formulations and are comparable to those reported for extrusion-based bioinks.^53^

Although bioactive glasses have been widely reported as modulators of gelation and rheological behavior in injectable hydrogels, their effects are highly system-dependent and strongly influenced by factors such as polymer chemistry, bioglass composition, particle size, concentration, and ion-release kinetics.^9^ In the present formulations, the viscosity–shear rate behaviors and the thixotropic recovery (3iTT) did not show statistically significant differences upon 58S bioglass incorporation. This behavior is consistent with a previous report indicating that bioglass addition does not necessarily alter shear-dependent viscosity or gel strength when polymer-driven network formation predominates.^54^ In systems dominated by physically cross-linked polymers, rheological behavior is primarily governed by polymer–polymer interactions, which may reduce the apparent contribution of B-mediated ionic effects. Moreover, increases in viscosity reported in the literature are often associated with time-dependent ionic interactions between released Ca^2+^ or Sr^2+^ ions and available carboxylate groups in the polymeric network.^55^ In the present system, the incorporation of 58S bioglass may similarly promote Ca^2+^ release and potential interactions with carboxylate groups from xanthan gum. However, under the conditions evaluated, such interactions were likely not sufficient to produce measurable differences in viscosity, possibly due to the dominance of polymer–polymer interactions and the physically cross-linked nature of the hydrogel network.

Overall, the rheological findings confirm that the developed hydrogels form a soft, physically cross-linked network governed by hydrophilic interactions. The pseudoplastic behavior, combined with high viscosity recovery (>80%) following high-shear deformation, particularly in bioactive glass-containing formulations, demonstrates the ability of the system to efficiently reorganize its structure. These favorable viscoelastic properties, characterized by controlled flow and rapid postinjection stabilization, indicate that these hydrogels are promising candidates for injectable carriers of bioactive agents in minimally invasive bone repair strategies.

#### 3.2.4 Performance Under Compression

Compression tests were performed to evaluate the influence of bioactive glass content on the mechanical properties of the hydrogels, with emphasis on Young’s modulus determination. Young’s modulus values were derived from the linear elastic region (5–20% strain) of the stress–strain curves presented in Figure 7, encompassing all evaluated replicates and formulations. The average Young’s modulus values were approximately 0.01 kPa for the three formulations, with no statistically significant differences according to Tukey’s test (Figure 8).

**Figure 7:**
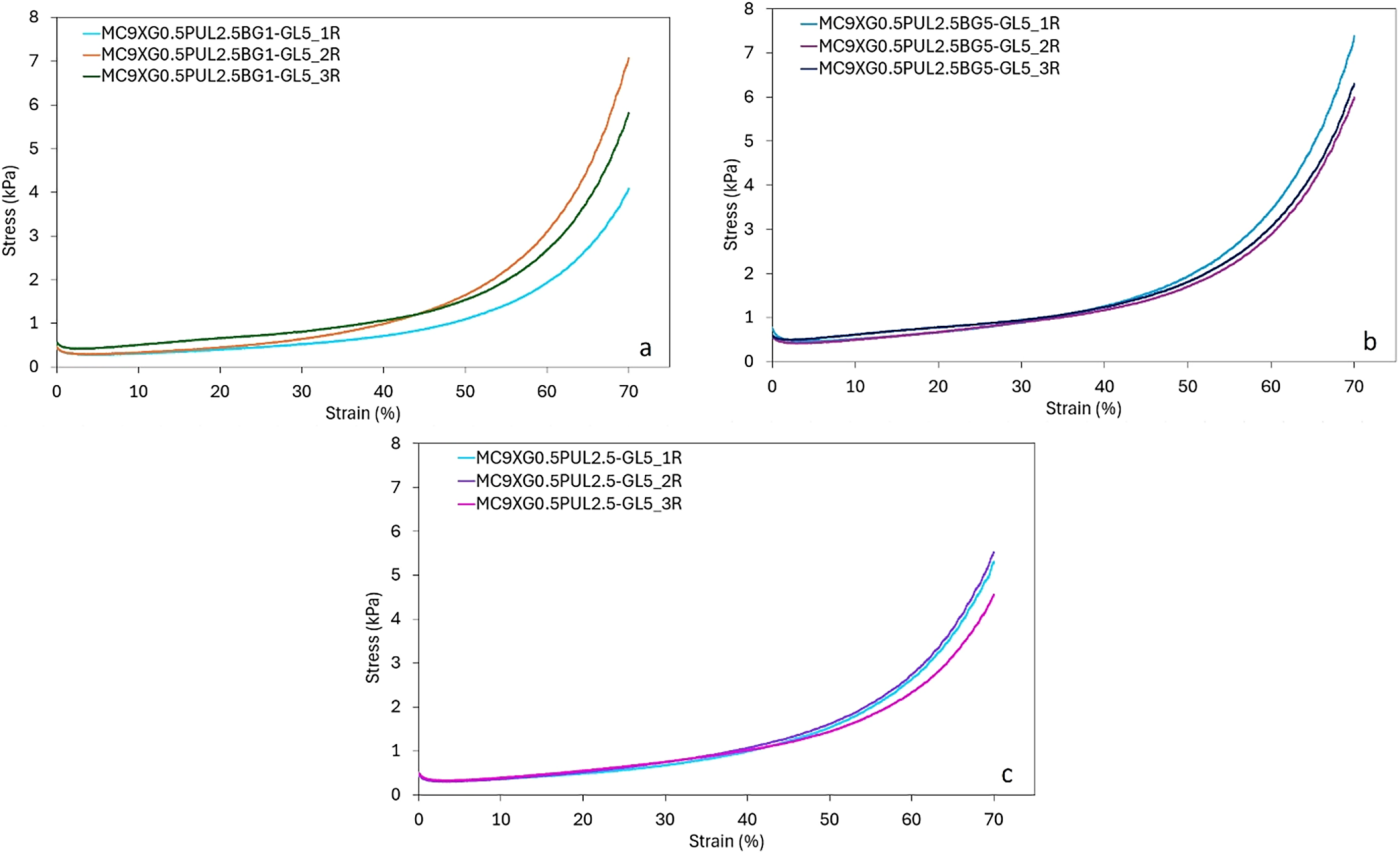
Compressive stress–strain curves for the ternary hydrogel formulations: (a) triplicate curves for MC9XG0.5PUL2.5BG1-GL5, (b) triplicate curves for MC9XG0.5PUL2.5BG5-GL5, and (c) triplicate curves for the control MC9XG0.5PUL2.5-GL5. Measurements were performed at 23 °C for samples previously gelled at 37 °C, using a crosshead speed of 0.1 mm/s up to 70% strain.

**Figure 8:**
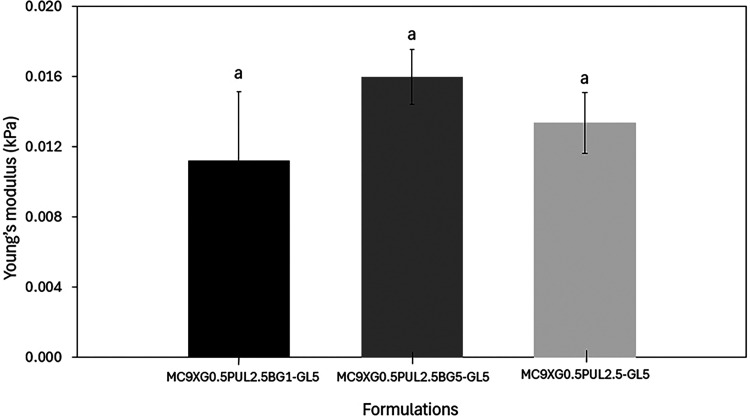
Young’s modulus of 5 to 20% deformation (linear region) of the formulations. The same letter indicates that there is no significant difference between the mean values (Tukey test, *p* < 0.05).

These results indicate that the hydrogels are extremely soft and highly deformable, a behavior characteristic of physically cross-linked polysaccharide-based networks with high water content. Similar low moduli have been reported for other polysaccharide hydrogels, even when chemically cross-linked. For instance, Zhang et al.^56^ reported Young’s moduli between 0.20 and 0.37 kPa for covalently cross-linked xanthan gum/silk fibroin hydrogels for tissue engineering and drug release applications, highlighting the dominant effect of hydration and loose network architecture on mechanical behavior.

In contrast, substantially higher compressive moduli have been observed in ionically cross-linked gellan gum hydrogels containing calcium-rich bioactive glasses, in which strong ionic interactions promote network stiffening.^57^ Although 58S bioglass releases divalent Ca^2+^ ions capable of interacting with xanthan gum carboxylate groups, the low xanthan gum content (0.5 wt %) in the present formulations likely limited the extent of ionic cross-linking and, consequently, the mechanical reinforcement of the addition of 58S bioglass.^32,41^

Furthermore, the remaining components also contribute to the low stiffness observed. Methylcellulose forms reversible physical networks stabilized by weak intermolecular interactions, while pullulan does not actively participate in network cross-linking. These features collectively favor soft, weakly structured hydrogels.^58,59^

The low Young’s modulus values are consistent with the rheological results, particularly the predominance of *G*″ over *G*′ in amplitude sweep tests and the frequency-dependent viscoelastic behavior, confirming the presence of a physically cross-linked network. From a biological perspective, material stiffness plays a critical role in cell response.^60^ While osteogenic differentiation is favored on substrates with higher stiffness, softer matrices are more suitable as injectable carriers and delivery systems.^61^

The mechanical results confirm that the developed hydrogels possess the essential characteristics for use as injectable bioactive carriers in nonload-bearing bone regeneration applications. This mechanical profile supports their application as specialized delivery systems that enable minimally invasive administration while allowing the 58S bioactive glass to effectively exert its osteoconductive function throughout the regenerative process.

#### 3.2.5 Swelling and Degradation

Swelling is a key parameter for hydrogels intended for biological applications, as it directly affects fluid uptake, nutrient diffusion, and the transport of bioactive agents. The swelling behavior of the formulations was evaluated in PBS after 24 h at 37 °C (Figure 9).

**Figure 9:**
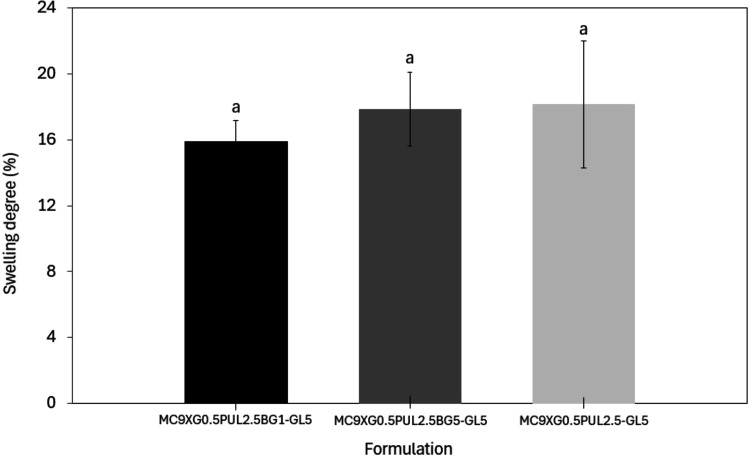
Absorption capacity of hydrogels MC9XG0.5PUL2.5-GL5, MC9XG0.5PUL2.5BG1-GL5, and MC9XG0.5PUL2.5BG5-GL5 in PBS after 24 h at 37 °C. The same letter indicates that there is no significant difference between the mean values (Tukey test, *p* < 0.05).

All formulations exhibited moderate swelling, ranging from 15.9% to 18.2% after 24 h. These values are consistent with previous reports for methylcellulose and xanthan gum–based hydrogels evaluated in PBS under similar conditions.^40^ Lower swelling in saline media is commonly attributed to the presence of ions that compete for hydration water, limiting polymer solvation.

In particular, methylcellulose is known to exhibit reduced hydration in ionic media due to a salting-out effect, in which chloride ions disrupt hydration cage structures around hydrophobic groups, promoting the formation of a more compact, physically cross-linked network.^62^ This effect has been reported to significantly reduce swelling in PBS compared to water.^32^ Additionally, xanthan gum, although highly hydrophilic, may experience partial charge screening in ionic environments, further restricting water uptake.^17^

The incorporation of bioactive glass further contributed to the reduced swelling observed. Ion exchange between 58S bioglass and PBS releases Na^+^ and Ca^2+^, increasing the ionic strength of the medium and intensifying the salting-out effect.^62^ Moreover, Ca^2+^ ions may partially neutralize xanthan gum carboxylate groups, decreasing electrostatic repulsion and limiting matrix expansion.^62^ Consequently, swelling values in the present study (∼16–18%) were lower than those reported for similar hydrogels without bioglass (around 30% in PBS).^32^ Despite this reduction, the swelling levels remain compatible with biological applications, allowing nutrient diffusion and metabolite transport within the hydrogel matrix.

Degradation behavior was also evaluated, as it is a key parameter for implantable hydrogels intended for bone regeneration, where degradation should be temporally compatible with tissue formation and bioactive agent release.^63^ Mass loss was monitored after incubation in PBS at 37 °C for 5, 10, 15, and 30 days (Figure 10).

**Figure 10:**
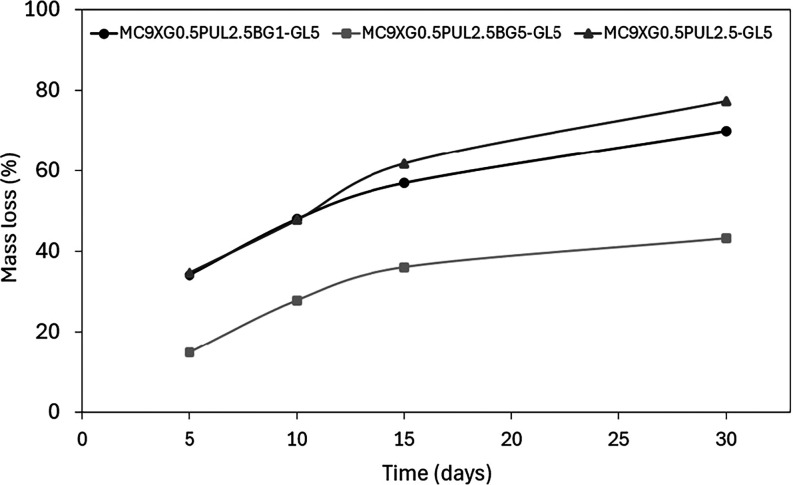
Graph of the mass loss kinetics of the formulations in PBS at 37 °C.

The results demonstrate a clear concentration-dependent effect of 58S bioglass on network stability. The control formulation showed the highest degradation (around 77% mass loss after 30 days), whereas formulations containing 1% and 5% 58S bioglass exhibited progressively lower mass loss, with the 5% formulation showing the greatest stability (about 43%). This behavior is attributed to ionic interactions between Ca^2+^ released from the bioglass and xanthan gum carboxylate groups, which contributed to increasing network cohesion and resistance to degradation.^41^

Similar stabilization effects induced by bioglass incorporation have been reported for other polysaccharide-based hydrogels, in which increased ionic cross-linking density reduces erosion rates.^48^ Thus, the present results indicate that 58S bioglass effectively modulates hydrogel degradation, with higher concentrations providing enhanced structural stability.

This concentration-dependent stabilization is particularly advantageous for bone regeneration strategies, as it enables the hydrogel to act as a bioactive carrier capable of maintaining the material at the implantation site during the early stages of bone repair.

## 4 Conclusion

Based on the results, the development of a multifunctional and thermoresponsive platform composed of methylcellulose, xanthan gum, and pullulan integrated with 58S bioglass represents an innovation for minimally invasive bone regeneration strategies. These hydrogels exhibit characteristics of smart injectable carriers, achieving *in situ* gelation at physiological temperatures and pseudoplastic behavior that facilitates precise extrusion into irregular bone defects.

Another innovation of this system is the dual role of the 58S bioglass, which functions as both an osteoconductive agent and a structural modulator by promoting ionic interactions between divalent cations (Ca^2+^) and the carboxylate groups of xanthan gum to create a stable network. This structural stability is further evidenced by a high thixotropic recovery above 80% and a tailored degradation profile, with the formulation incorporating 5% 58S bioglass showing significantly lower mass loss compared to the control.

While SEM analysis revealed a constricted macroporous architecture between 18 and 30 μm, this characterization reflects a static snapshot in the lyophilized state rather than the dynamic behavior of the matrix in physiological environments. In such conditions, the moderate swelling capacity and controlled degradability of the material ensure a dynamic evolution of the structure, which progressively generates space for tissue ingrowth and cellular signaling.

Mechanically, the formulations are characterized as ultralow-stiffness materials (E approximately 0.01 kPa), confirming their suitability as bioactive carriers that prioritize localized ionic delivery and biological signaling over structural load-bearing. Combined with preliminary noncytotoxicity confirmed via indirect assays, the developed system emerges as a versatile and safe platform for further investigation in bone repair. Ultimately, the synergy between the ternary polymeric blend and the 58S bioglass, combining injectability, thermoresponsivity, and biological safety, positions this system as a promising potential solution for the treatment of non-load-bearing bone defects, paving the way for future detailed *in vitro* and *in vivo* studies.

## Supplementary Material



## Notes

Temperature-dependent gelation behavior assessed by qualitative analysis (Figure S1); FTIR spectral analysis of the hydrogel formulations (Figure S2), including band assignments and discussion; thermogravimetric analysis (TGA) of the hydrogel formulations, including thermal decomposition curves (Figure S3) and discussion (PDF)
