# Supplementary material for: Characterization of Thermoresponsive Methylcellulose-Based Injectable Hydrogels Incorporating 58S Bioactive Glass for Non-Load-Bearing Bone Regeneration
Source: ACS Omega. 2026 May 14;11(20):29843–57. doi: 10.1021/acsomega.6c00586 (PMC13216992; doi:10.1021/acsomega.6c00586)
Supplement: Supplementary file 1 [file ao6c00586_si_001.pdf]

**Supporting Information**

# Characterization of Thermoresponsive Methylcellulose-Based Injectable Hydrogels Incorporating 58S Bioactive Glass for Non-Load-Bearing Bone Regeneration

*Marina Bosso, Ângela Maria Moraes**

*Department of Engineering of Materials and of Bioprocesses/School of Chemical Engineering/University of Campinas – Av. Albert Einstein 13083-852 Campinas SP – Brazil*

**Corresponding author: ammoraes@unicamp.br*

# 1 RESULTS AND DISCUSSION

## 1.1 Selection of Adequate Formulations Regarding Gelation Temperature and Injectability

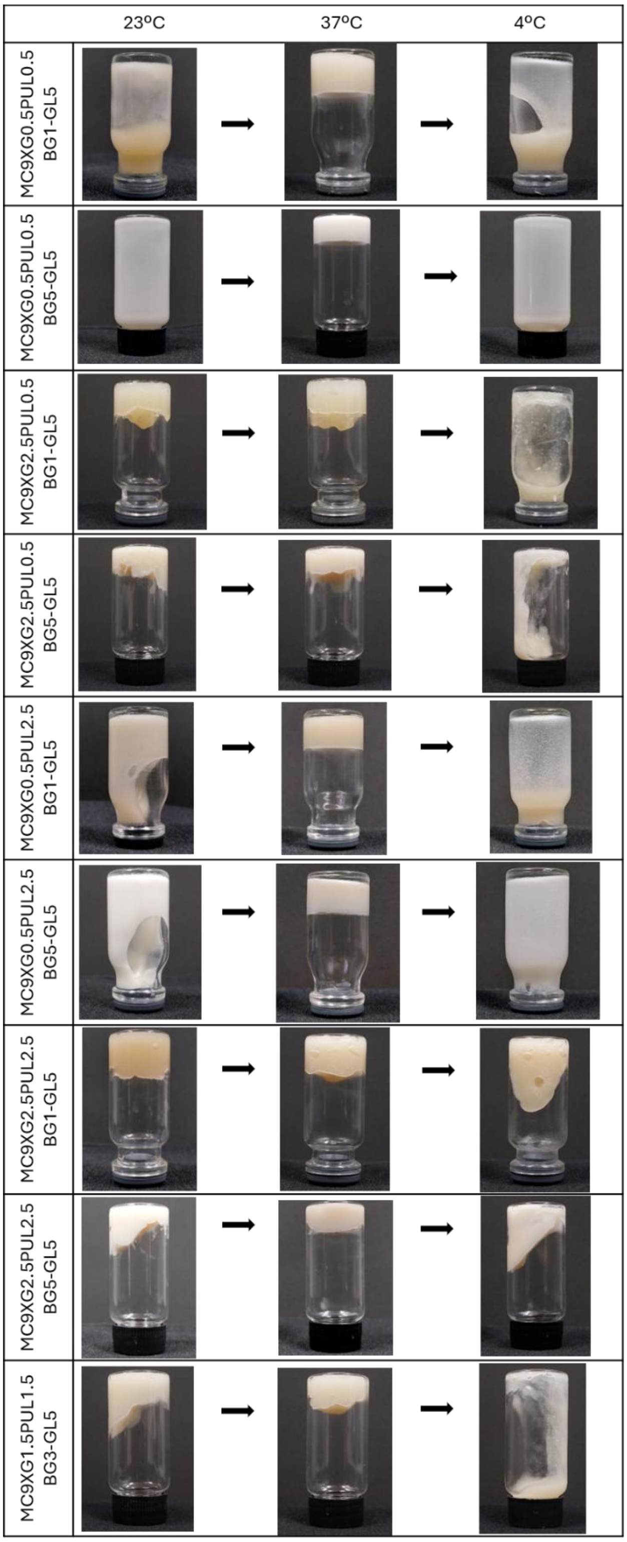


Figure S1. Flowability assessment of the formulations at varying temperatures using the tube inversion method.

## 1.2 Fourier transform infrared spectroscopy (FTIR)

The chemical structure of the hydrogels was investigated by FTIR to identify functional groups and possible interactions among the polymeric components and the bioglass. The spectra of the formulations MC9XG0.5PUL2.5-GL5, MC9XG0.5PUL2.5BG1-GL5, and MC9XG0.5PUL2.5BG5-GL5, as well as those of the individual components (methylcellulose, xanthan gum, pullulan, and 58S bioglass), are shown in Figure S2.

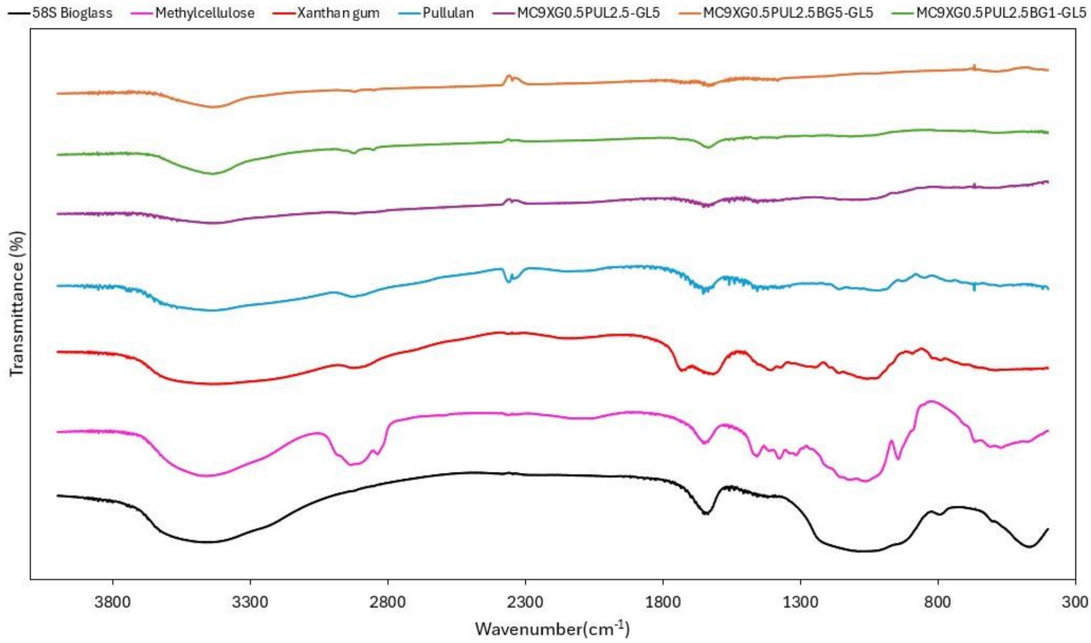


Figure S2. FTIR spectra of the isolated components methylcellulose, xanthan gum, pullulan, bioglass and the hydrogels MC9XG0.5PUL2.5-GL5, MC9XG0.5PUL2.5BG1-GL5, and MC9XG0.5PUL2.5BG5-GL5.

All biopolymers exhibited similar spectral features, particularly methylcellulose and pullulan, reflecting their polysaccharide nature. The spectra of all formulations showed a broad O–H stretching band between 3400 and 3300 $cm^{-1}$, associated with hydroxyl groups and hydrogen bonding, typical of hydrophilic polymeric systems[1]. In xanthan gum, this band appeared broader, consistent with the presence of carboxylic acid groups[1,2]. Additional common bands included C–H stretching of $sp^3$ carbons (3000–2840 $cm^{-1}$), $CH_3$ bending around 1375 $cm^{-1}$, and C–O stretching of primary alcohols between 1100 and 1070 $cm^{-1}$ [1,3].

Methylcellulose presented characteristic ether-related bands, notably the aliphatic C–O–C stretching near 1120 $cm^{-1}$ [3,4]. Xanthan gum exhibited additional signals associated with carboxylic groups, including bands between 1600 and 1450 $cm^{-1}$ and C=O stretching in the 1730–1700 $cm^{-1}$ range[2,4]. Pullulan showed a pronounced C–O stretching band between 1260 and 1000 $cm^{-1}$, consistent with its high density of hydroxyl groups[5].

The 58S bioglass spectrum displayed bands related to adsorbed water (1700–1600 $cm^{-1}$), asymmetric Si–O–Si stretching (1200–1000 $cm^{-1}$), and symmetric Si–O–Si stretching around 820–800 $cm^{-1}$ [6]. Phosphate-related bands were not detected, likely due to their low concentration in the glass composition.

The FTIR spectra of the hydrogel formulations were highly similar to each other and predominantly reflected the spectral features of the polymeric matrix. Characteristic bands of hydroxyl, aliphatic C–H, and C–O groups were preserved in all formulations[6,7]. The absence of new vibrational bands indicates that no additional covalent bonds or significant chemical crosslinking were formed during hydrogel preparation. Moreover, the lack of clearly distinguishable bioglass bands in the composite spectra may be attributed to its low concentration and homogeneous dispersion within the polymeric matrix, limiting direct FTIR detection.

## 1.3 Thermogravimetric analysis (TGA)

Thermogravimetric analysis was performed as a standard thermal characterization to investigate the thermal degradation behavior of the hydrogels and to assess their stability under autoclave sterilization temperature (121 °C), which is required for implantable medical products. The TGA and DTG curves of the lyophilized formulations are shown in Figure S3.

All formulations exhibited three main thermal events, characteristic of hydrophilic polymeric systems and polymer–inorganic composites. The first event occurred at relatively low temperatures (around 68–80 °C), which was attributed to the loss of free and weakly bound water retained within the hydrogel network due to the high density of hydroxyl groups from methylcellulose, pullulan, and xanthan gum. The associated mass

loss was low (around 2–3%) and consistent with previous reports for methylcellulose- and pullulan-based hydrogels, in which dehydration typically occurs below 100 °C [8,9].

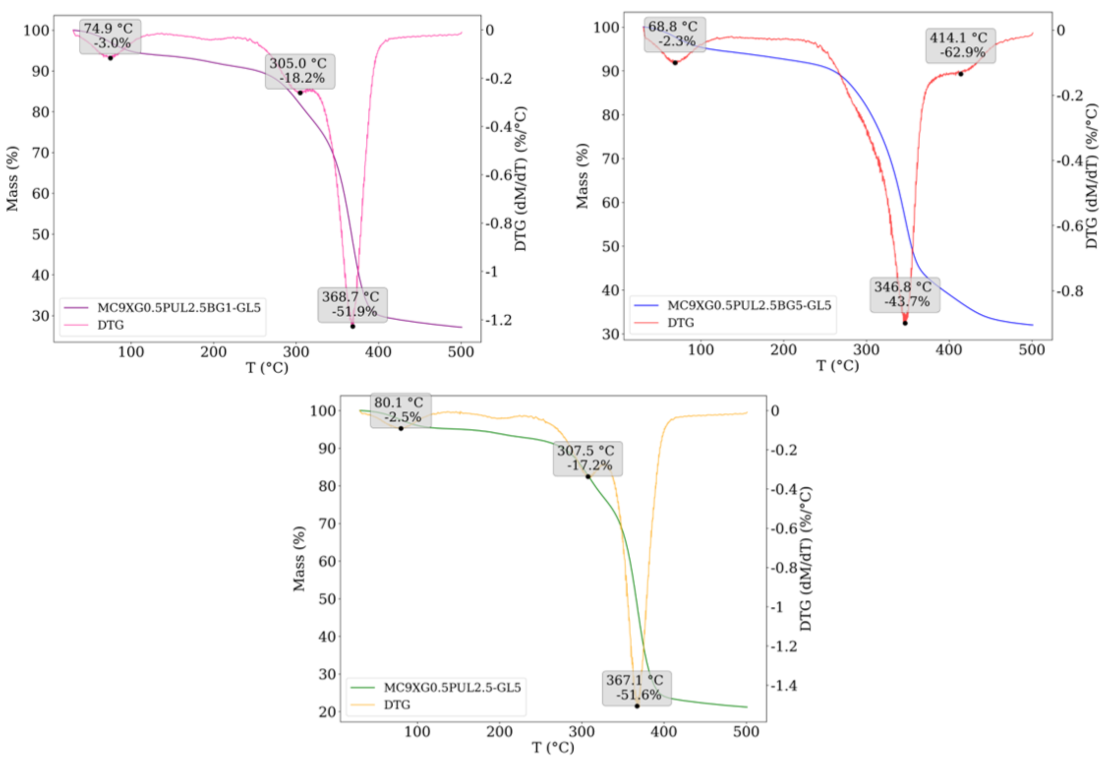


Figure S3. Thermogravimetric (TG) and derivative thermogravimetric (DTG) curves, with indications of the degradation peaks for each of the formulations: MC9XG0.5PUL2.5BG1-GL5; MC9XG0.5PUL2.5BG5-GL5, and MC9XG0.5PUL2.5-GL5.

The second thermal event, observed between approximately 305 and 347 °C, corresponded to the main degradation of the polymeric backbone, involving the cleavage of C–O–C and C–C bonds and the release of volatile products such as CO and $CO_2$. This degradation range aligns with literature data for methylcellulose, pullulan, xanthan gum, and related polysaccharide-based hydrogels[3,8,9]. Notably, the formulation containing 5% 58S bioglass exhibited a higher degradation temperature (around 347 °C) and a larger mass loss in this stage, suggesting enhanced thermal stability. This behavior is attributed to interactions between bioglass particles and polymer hydroxyl groups, which may restrict chain mobility and reduce the mass loss[10].

The third and most intense degradation event occurred at higher temperatures (between 367 and 414 °C) and was associated with the decomposition of more stable carbonaceous residues and the combustion of the remaining organic matter. Similar high-

temperature degradation stages have been reported for pullulan-based systems and polysaccharide composites containing inorganic phases, for which the presence of bioglass alters heat diffusion and degradation dynamics[8,10].

It is noteworthy that no significant thermal degradation was observed at 121 °C for any formulation. The initial mass losses observed below this temperature are consistent with dehydration phenomena. No mass loss events attributable to thermal decomposition of the polymer backbone were detected within this temperature range, suggesting adequate thermal resistance of the hydrogels under autoclave sterilization conditions. Furthermore, the incorporation of 58S bioglass, particularly at higher concentrations, contributed to improved thermal stability at elevated temperatures, reinforcing the suitability of these formulations for implantable applications requiring thermal sterilization before clinical use.

## 2 REFERENCES

(1) Pavia, D.; Lampman, G.; Kriz, G.; Vyvyan, J. *Introdução à espectroscopia*; Cengage Learning, 2015.

(2) Faria, S.; De Oliveira Petkowicz, C. L.; De Morais, S. A. L.; Terrones, M. G. H.; De Resende, M. M.; De França, F. P.; Cardoso, V. L. Characterization of Xanthan Gum Produced from Sugar Cane Broth. *Carbohydrate Polymers* **2011**, *86* (2), 469–476.

(3) Oliveira, R. L.; Vieira, J. G.; Barud, H. S.; Assunção, R. M. N.; Rodrigues Filho, G.; Ribeiro, S. J. L.; Messadeqq, Y. Synthesis and Characterization of Methylcellulose Produced from Bacterial Cellulose under Heterogeneous Condition. *Journal of the Brazilian Chemical Society* **2015**.

(4) Liu, Z.; Yao, P. Injectable Thermo-Responsive Hydrogel Composed of Xanthan Gum and Methylcellulose Double Networks with Shear-Thinning Property. *Carbohydrate Polymers* **2015**, *132*, 490–498.

(5) Haghighatpanah, N.; Mirzaee, H.; Khodaiyan, F.; Kennedy, J. F.; Aghakhani, A.; Hosseini, S. S.; Jahanbin, K. Optimization and Characterization of Pullulan Produced by a Newly Identified Strain of Aureobasidium Pullulans. *International Journal of Biological Macromolecules* **2020**, *152*, 305–313.

(6) Moreira, C. D. F.; Carvalho, S. M.; Sousa, R. G.; Mansur, H. S.; Pereira, M. M. Nanostructured Chitosan/Gelatin/Bioactive Glass in Situ Forming Hydrogel Composites as a Potential Injectable Matrix for Bone Tissue Engineering. *Materials Chemistry and Physics* **2018**, *218*, 304–316.

(7) Westin, C. B. Desenvolvimento de hidrogéis poliméricos termossensíveis contendo agentes bioativos para engenharia de tecido cartilaginoso: **Thesis**, University of Campinas (UNICAMP), Campinas, 2020.

(8) Su, T.; Wu, L.; Pan, X.; Zhang, C.; Shi, M.; Gao, R.; Qi, X.; Dong, W. Pullulan-Derived Nanocomposite Hydrogels for Wastewater Remediation: Synthesis and Characterization. *Journal of Colloid and Interface Science* **2019**, *542*, 253–262.

(9) Vijayaraghavan, R.; Loganathan, S.; Valapa, R. B. 3D Bioprinted Photo Crosslinkable GelMA/Methylcellulose Hydrogel Mimicking Native Corneal Model with Enhanced in Vitro Cytocompatibility and Sustained Keratocyte Phenotype for Stromal Regeneration. *International Journal of Biological Macromolecules* **2024**, *264*, 130472.

(10) Ferreira, F. V.; Souza, L. P.; Martins, T. M. M.; Lopes, J. H.; Mattos, B. D.; Mariano, M.; Pinheiro, I. F.; Valverde, T. M.; Livi, S.; Camilli, J. A.; Goes, A. M.; Gouveia, R. F.; Lona, L. M. F.; Rojas, O. J. Nanocellulose/Bioactive Glass Cryogels as Scaffolds for Bone Regeneration. *Nanoscale* **2019**, *11* (42), 19842–19849.
